# Supplementary material for: CIGAR‐seq, a CRISPR/Cas‐based method for unbiased screening of novel mRNA modification regulators
Source: Mol Syst Biol. 2020 Nov 30;16(11):e10025. doi: 10.15252/msb.202010025 (PMC7701898; doi:10.15252/msb.202010025)
Supplement: Supplementary file 1 — Expanded View Figures PDF [file MSB-16-e10025-s001.pdf]

Expanded View Figures

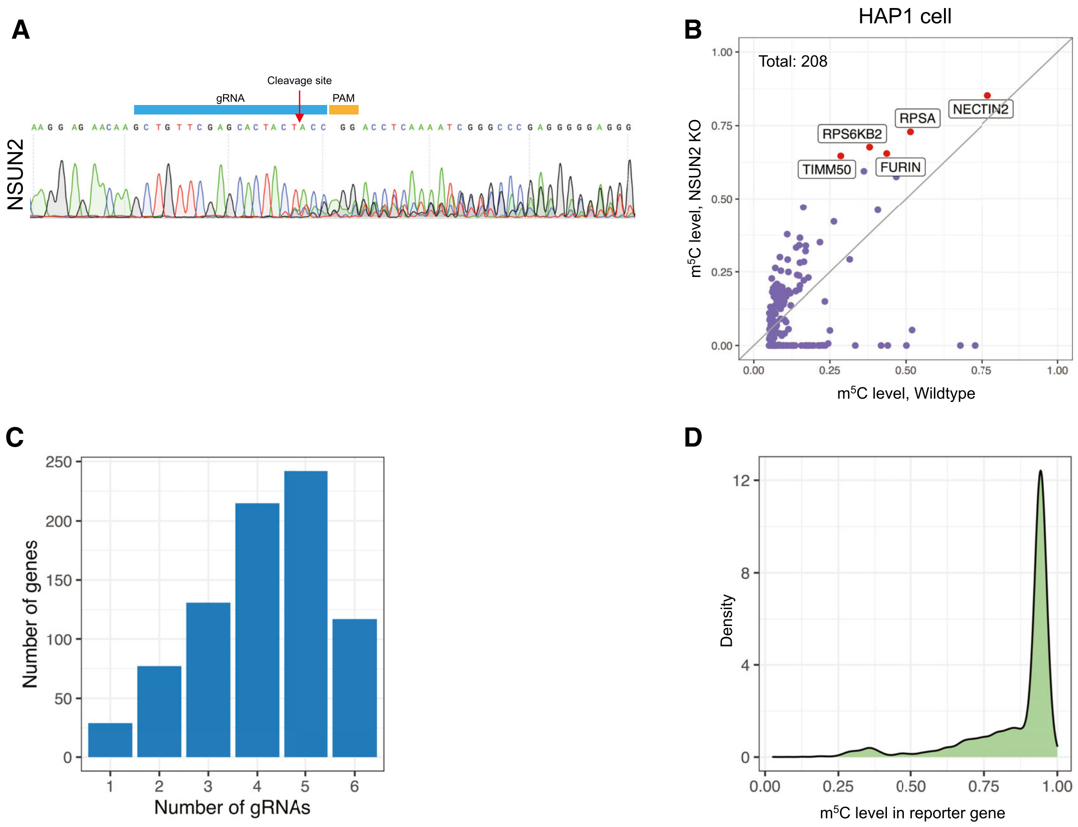

Figure EV1. NSUN2 Knockout and the effect on the reporter as well as endogenous m^5C sites.

A The NSUN2-KO efficiency in Cas9-expressing HAP1 cells. Seven days after viral transduction, NSUN2 was efficiently mutated.

B Scatter plot demonstrating the effect of NSUN2 knocked out in HAP1 cells. 208 m^5C sites were identified in wild-type HAP1 cells, 90 (43.3%) of which showed significantly reduced m^5C level in NSUN2-KO cells. The five m^5C sites with the highest modification rate in NSUN2-KO cells were highlighted as red dots and marked with gene names. The gray line represents the diagonal line, along which the modification rate is equal between wild-type and NSUN2 knockout cells.

C Distribution of genes with different number of gRNAs detected.

D Density plot of m^5C level of the reporter site associated with individual gRNA. The Y-axis represents the density of gRNAs.

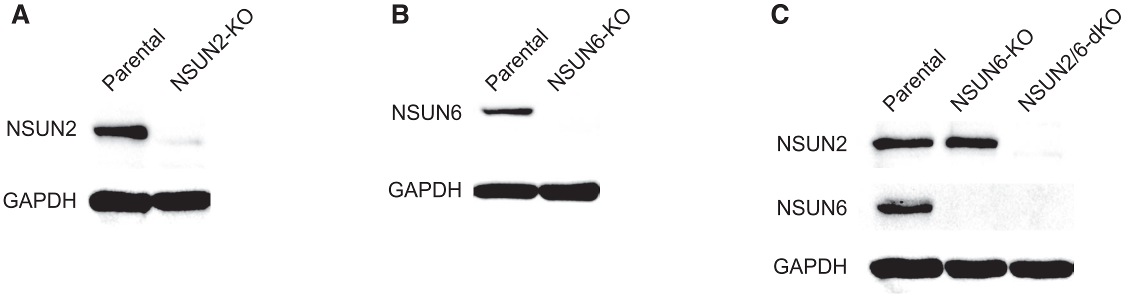

Figure EV2. KO of NSUN2 and/or NSUN6 in HAP1 cells.

A–C Western Blot demonstrating the effect NSUN2 (A), NSUN6 (B) as well as NSUN2/6 double knockout (C). NSUN2/6 double knockout (NSUN2/6-dKO) HAP1 cells were established based on clonal NSUN6-KO cells.

© 2020 The Authors

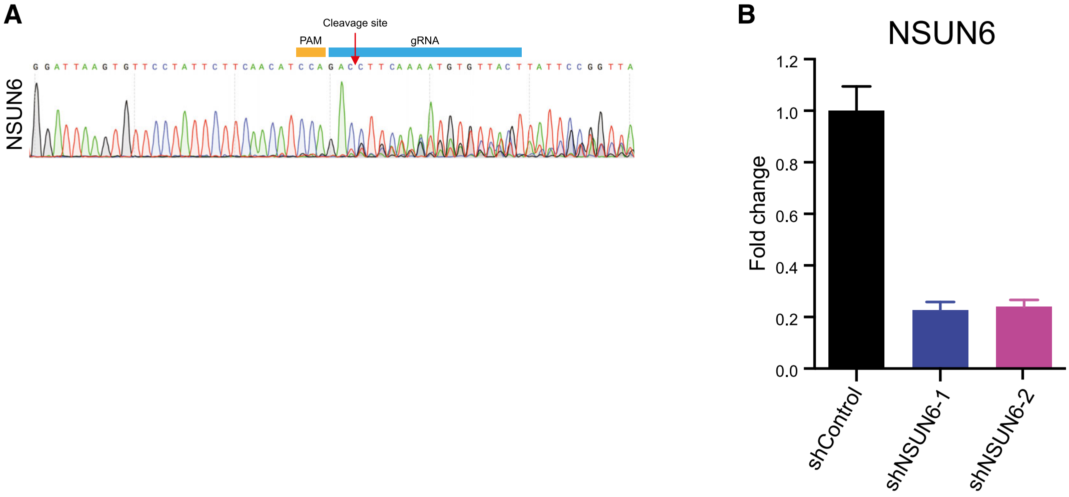

Figure EV3. NSUN6 was efficiently knocked out or knocked down.

A The KO efficiency of NSUN6 in Cas9-expressing HAP1 cells. Seven days after viral transduction, NSUN6 was efficiently mutated.

B The knockdown efficiency of NSUN6 in HAP1 cells. Three independent biological replicates were performed. Error bars represent SD.

Figure EV4. mRNA-BisSeq profiles of the three m^{5}C modification sites depend on both NSUN6 and NSUN2.

A–C IGV plots showing the m^{5}C sites in ANGEL1, ZNF707, and STRN4 genes. The m^{5}C sites in ANGEL1 (A) and ZNF707 (B) possessed a 3′ TCCA and a 3′ AGGG motif, respectively, while the m^{5}C site in STRN4 was among a cluster of “pseudo” m^{5}C sites (C).

© 2020 The Authors

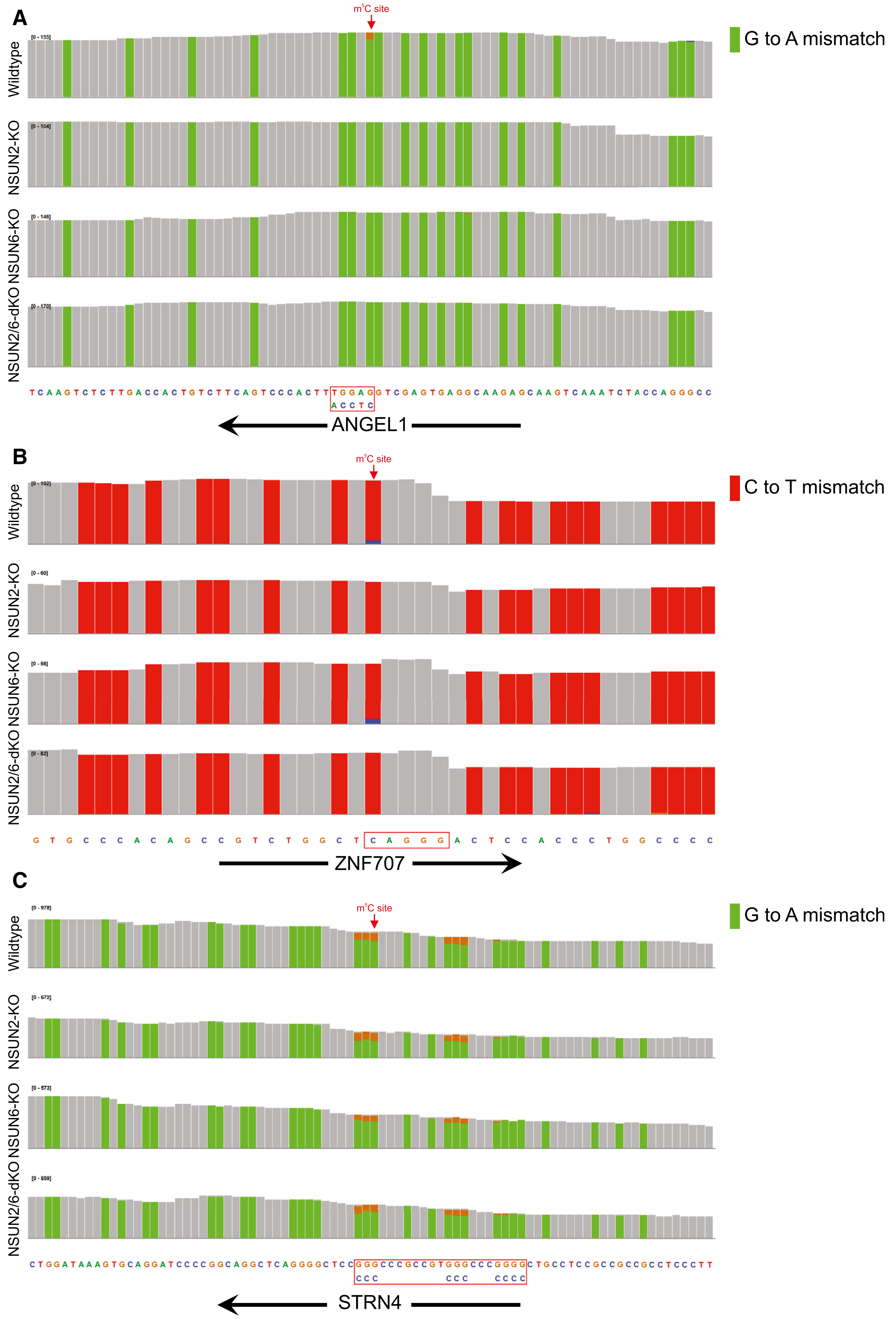
A
m5C site
Wildtype
NSUN2-KO
NSUN6-KO
NSUN2/6-dKO
G to A mismatch
ANGEL1
B
m5C site
Wildtype
NSUN2-KO
NSUN6-KO
NSUN2/6-dKO
C to T mismatch
ZNF707
C
m5C site
Wildtype
NSUN2-KO
NSUN6-KO
NSUN2/6-dKO
G to A mismatch
STRN4

Figure EV4.

ID	position	gene	sequences
1	11@16756661@+	C11orf58	cacacctataatcccagcactttgggaggccgaggtg
2	11@66687583@-	SPTBN2	GCATTGCAGATGGGGCCGGGCCCCGAGTCCGGGTC
3	11@767963@-	GATD1	gggttcaagcgattctcctgcctcagcctcccaag
4	11@767969@-	GATD1	gggttcaagcgattctcctgcctcagcctcccaag
5	11@768098@-	GATD1	cctcaggtgatccacctgcctcagcctcccaaagtg
6	11@768104@-	GATD1	cctcaggtgatccacctgcctcagcctcccaaagtg
7	12@110281749@+	ATP2A2	CCCGCGGCCGgagtgcgaggcggaggcgaggaggccgcg
8	12@110281750@+	ATP2A2	CCCGCGGCCGgagtgcgaggcggaggcgaggaggccgcg
9	12@110281752@+	ATP2A2	CCCGCGGCCGgagtgcgaggcggaggcgaggaggccgcg
10	14@74736311@+	FCF1	cccagccactggggaggctgaggcaggagaacctctt
11	14@74736317@+	FCF1	cccagccactggggaggctgaggcaggagaacctctt
12	15@65150178@-	CLPX	gcgattctcctgcctcagcctcccgagtagctgggat
13	15@85234697@-	AC044860.1	CAGCCACCCCGCGCACCGGCTACAAGCCGCCCGGGGG
14	16@1964562@-	RPS2	CCGCGGAAGCCACCGCGGTTCCCCATCCCAGGGCC
15	16@2972795@+	PAQR4	AGGAAGGGCCATGTTGCCACATGAGCAAGCTTGGGTG
16	16@50368785@-	BRD7	GCCCATGTCCGAccgggcccggtgcccgccccccg
17	18@31685103@-		ATGCCGCGCCGGGCCCCGGCGCCCCGCGGCCGCCGCG
18	19@13118331@+	NACC1	CGCCGCGGCTGCCGCTGCTgctgaggcggaggccgcggaggccg
19	19@13118340@+	NACC1	CGCCGCGGCTGCCGCTGCTgctgaggcggaggccgcggaggccg
20	1@150308596@+	MRPS21	cccaacactttgggaggctgaggcgggcggctcact
21	1@156676704@-	NES	GGTAGCCGCGCACTGCCCCGCGCCACGCCTCGCCCA
22	1@31938230@-	PTP4A2	tcctgctgccgccgctgcctccgctgccgccgctg
23	20@36891800@-	SAMHD1	gttcgagcaatcctcctgcctcagcctcccaagtagc
24	20@43667277@+	MYBL2	CGCGGCGCGGTCCGGGCCGGGGGATGTCTCGGCGGA
25	22@49961503@+	PIM3	GGGCGCGGCGGGCGGCGCGCGCGGCGTCATCCGCCT
26	2@101253124@+	CNOT11	GGGTCCGGGAGCGGAGGCCCGGGGGGCCCCCGCGGG
27	2@216633613@+	IGFBP2	ctgggcgcgagtggcggcggcggcggggcgcgcgCG
28	2@216633616@+	IGFBP2	ctgggcgcgagtggcggcggcggcggggcgcgcgCG
29	2@216633619@+	IGFBP2	ctgggcgcgagtggcggcggcggcggggcgcgcgCG
30	5@180071683@-	RNF130	GAGCCGGGCAGGGCCCGCCCGCCCCGCGCAGCTCA
31	5@180071687@-	RNF130	GAGCCGGGCAGGGCCCGCCCGCCCCGCGCAGCTCA
32	6@113859942@+	MARCKS	CCCGGCTCGCCCACGGCCGCGGAGGGAGAGGCCGC
33	6@113859943@+	MARCKS	CCCGGCTCGCCCACGGCCGCGGAGGGAGAGGCCGC
34	6@113859945@+	MARCKS	CCCGGCTCGCCCACGGCCGCGGAGGGAGAGGCCGC
35	6@149724702@-	NUP43	gcaattctcctgcctcagcctcccaagtagctgggat
36	7@100070158@-	ZNF3	TCTGGGCTTCGtttttttgtttttttgttttttttt
37	7@100070166@-	ZNF3	TCTGGGCTTCGtttttttgtttttttgttttttttt
38	X@119538511@-	CXorf56	gcaatctcagctcactgcaacctccacctcccgagt
39	X@119538527@-	CXorf56	gcaacctccacctcccgagttcaagcaattctcctg
40	7@151080695@-	FASTK	CCCTACATGACTCCCCGGGCCCTGCGCAGGTCGCTCC
41	7@151080696@-	FASTK	CCCTACATGACTCCCCGGGCCCTGCGCAGGTCGCTCC
42	7@151080697@-	FASTK	CCCTACATGACTCCCCGGGCCCTGCGCAGGTCGCTCC
43	8@12755322@-	LONRF1	TCCAGCCGATGGCCGCTGCCGCCGCCCACTTCCCAG
44	8@17156688@+	ZDHHC2	CCAGGCCCGCGGGCGGCGGCGGAGCTGGGCAGGTG
45	8@17156691@+	ZDHHC2	CCAGGCCCGCGGGCGGCGGCGGAGCTGGGCAGGTG
46	8@17156694@+	ZDHHC2	CCAGGCCCGCGGGCGGCGGCGGAGCTGGGCAGGTG
47	MT@14423@-	MT-ND6	CAAGACCTCAACCCCTGACCCCCATGCCTCAGGAT
48	MT@1486@+	MT-RNR1	AAGCGCGTACACACCGCCCGTCACCCTCCTCAAGT
49	MT@1488@+	MT-RNR1	AAGCGCGTACACACCGCCCGTCACCCTCCTCAAGT
50	MT@8387@-		CTAAATACTACCGTATGGCCCACCATAATTACCCC
51	MT@8391@-		CTAAATACTACCGTATGGCCCACCATAATTACCCC
52	MT@8392@-		CTAAATACTACCGTATGGCCCACCATAATTACCCC
53	6@42989628@+	**PPP2R5D**	GAGCCCCCCAAGGTTGCCAAATGCACAGCCAAGCCTA
54	7@134293043@-	**SLC35B4**	ATCAGAGAATAGCTCTGGACAGTGGAATAAACATAC
55	5@179616893@-	**HNRNPH1**	ATGCCTCCCATCATTTGGCTACCATAAGCACCACC
56	5@80626854@-	**DHFR**	accaaccctttccccttgtttttttttttTTTTTTTT

Figure EV5. NSUN2/6-independent m^{5}C sites.

The group of 56 NSUN2/6-independent sites was highly enriched for clusters of pseudo m^{5}C sites: 52 sites had at least one pseudo m^{5}C site in vicinity.

© 2020 The Authors
